# Influence of time-of-day on neuromuscular performance in team sport athletes: a systematic review and meta-analysis

**DOI:** 10.3389/fspor.2024.1466050

**Published:** 2025-01-17

**Authors:** Julio Martin-López, Alberto Pérez-López, David Varillas-Delgado, Álvaro López-Samanes

**Affiliations:** ^1^ Faculty of Health Sciences, School of Sports Sciences, Universidad Francisco de Vitoria, Madrid, Spain; ^2^ Departamento de Ciencias Biomédicas, Área de Educación Física y Deportiva, Facultad de Medicina y Ciencias de la Salud, Universidad de Alcalá, Madrid, Spain; ^3^ GICAF Research Group, Education, Research Methods and Evaluation Department, Faculty of Human and Social Sciences, Universidad Pontificia Comillas, Madrid, Spain

**Keywords:** time-of-day, neuromuscular performance, team-sports, power, agility

## Abstract

**Introduction:**

Although circadian rhythms have been shown to influence some neuromuscular performance tasks, the time-of-day effect on team sports performance athletes remains equivocal. This study aimed to examine the existing evidence concerning diurnal variations in neuromuscular performance in professional and semi-professional team sports athletes using a meta-analytic approach.

**Methods:**

A literature search was conducted through three different databases: PubMed, SportDiscus and Web of Science. Article selection was made based on the following inclusion criteria: team sports athletes, professional or semi-professional athletes, neuromuscular performance, testing protocols and time-of-day testing times. Neuromuscular performance parameters such vertical jump capacity (i.e., squat and countermovement jump), agility and isometric strength were included in the analysis. Testing protocols that specifically assessed these parameters across morning (AM) and late afternoon/evening (PM) periods were considered were extracted from the selected studies.

**Results:**

Ten studies met the inclusion criteria for qualitative synthesis and five for quantitative synthesis. Meta-analysis indicated lower countermovement jump in the AM compared to with PM (mean difference, −1.44; 95% CI −2.80 to −0.08; *p* = 0.04) and higher agility performance (mean difference 0.42; 95% CI 0.09–0.74; *p* = 0.01) in PM comparing with AM. No differences were reported in isometric strength and squat jump performance (*p* > 0.05).

**Conclusion:**

Neuromuscular performance is higher in the late afternoon or early evening compared to morning schedules in team sport athletes. Hence, time-of-day variations need to be considered when evaluating neuromuscular performance in professional and semi-professional team sports athletes.

## 1 Introduction

In team sport athletes, the coordination of muscle recruitment patterns and the control of movements through proprioceptive feedback reflect the efficacy of the neuromuscular system in initiating and directing specific sports actions that determine performance (1, 2). Several aspects could impact neuromuscular performance including nutritional status (3), hormonal fluctuations (4), muscle fiber typology (5), or time-of-day when training/competitions are developed (6). Referring to the effect of time-of-day, it is worth mentioning the last years exponential increase in the number of scientific investigations that analyzed their impact on neuromuscular performance despite limited evidence in professional and semi-professional athletes can be found (7).

Numerous factors have been proposed to elucidate the potential time-of-day variations in neuromuscular performance such as external factors (i.e., ambient conditions), lifestyle conditions (e.g., time awake and times of training and habitual competition) or internal (endogenous) factors such as chronotype (i.e., manifestation of circadian rhythmicity and predispositions towards morning or evening orientation) or hormonal fluctuations (e.g., cortisol/melatonin) (8, 9). Regarding hormonal rhythms, particularly cortisol and melatonin, play a crucial role in influencing athletic performance by regulating energy levels, alertness, and recovery. Cortisol levels peak in the morning, enhancing physical readiness (10), while melatonin rises at night, facilitating recovery but potentially hindering late-evening performance (11). Athletes' chronotypes, whether they are naturally inclined toward early or late activity, interact with these hormonal cycles, with early chronotypes excelling in the morning and late chronotypes performing optimally in the evening (10, 11). Aligning training and competition schedules with an athlete's chronotype, alongside strategies such as sleep regulation and light exposure, can significantly enhance both performance and recovery, improving mood and cognitive function. In addition to physiological factors, circadian timing affects mood and cognitive function, which are essential for decision-making and situational awareness in team sports. Group-based exercise at specific times has been shown to improve mood and reduce depressive symptoms (12). Furthermore, morning exercise has been found to counteract cognitive impairments caused by partial sleep deprivation offering practical insights for optimizing athletic performance, especially under challenging or irregular schedules (13). However, the majority of the studies published on this topic have been developed on endurance (e.g., swimming or cycling) (14, 15) or power/strength disciplines (16, 17), existing limited evidence in specific tasks of team-sports athletes.

The identification of time-of-day fluctuations in neuromuscular performance among team-sports athletes may provide useful information for coaches and performance staff to schedule training and competitions. Nevertheless, a reduced number of studies have been developed in team-sports athletes and most of them are developed in young athletes (18, 19) or amateur/recreational team sports players (20, 21), with only a few focusing on professional and semi-professional players engaged in team sports athletes (22, 23). Since minimal differences in performance (≈1%) might determine success in high-level athletes (24) the understanding of the potential time-of-day influence on neuromuscular performance would contribute to maximizing professional and semi-professional performance. Hence, the aim of this systematic review and meta-analysis was to examine the time-of-day effect in neuromuscular performance (e.g., vertical jump or agility capacity) on professional or semi-professional team sports athletes. Based on previous findings we hypothesized that neuromuscular performance related to team sports disciplines will be less pronounced in the morning in comparison to late afternoon and early evening in professional and semi-professional team sports athletes.

## 2 Methods

The entire systematic review and meta-analysis process followed the guidelines outlined in the Preferred Reporting Items for Systematic Review and Meta-Analyses (PRISMA) (25). The review was registered in July 2022 and updated in November 2023 on PROSPERO (ID = CRD42022347351).

### 2.1 Search strategy, information sources and data managements

A detailed search of the literature was explored using the databases Medline (PubMed), SPORTDiscus, and Web of Science using all the available articles until November 2023. The search strategy was designed to explore areas related to circadian rhythms, chronotype and sports performance in professional and semi-professional sports teams players using a combination of the following keywords: (“professional player” OR “sports player” OR “high-performance player” OR “semi-professional player” OR “soccer players” OR “football players” OR “basketball players” OR “baseball players” OR “hockey players” OR “handball players” OR “rugby players” OR “volleyball players” OR “elite players” OR “athlete”) AND (“time of day” OR “time-of-day” OR “morning” OR “evening” OR “morningness-eveningness”, OR “circadian rhythms” OR “chronobiology” OR “chronotype”) AND (“performance” OR “physical performance” OR “exercise performance” OR “sports performance” OR “neuromuscular performance” OR “body temperature”).

### 2.2 Eligibility criteria

The PICOS model (26) was utilized to establish the inclusion criteria being (a) studies carried out in team sports athletes; (b) professional or semi-professional team sports athletes; (c) studies comparing the morning vs. later afternoon or early evening performance on team sports athletes; (d) studies with experimental designs, specifically randomized crossover trials, that assessed neuromuscular performance in controlled conditions. Exclusion criteria were: (a) studies involving individual sports players; (b) amateur/recreational athletes; (c) jetlag or sleep deprivation studies; (d) studies that applied any dietary supplement (e.g., melatonin) that may affect sports performance; (e) retrospective studies.

### 2.3 Data collection process and data items

Two researchers independently extracted data for each study that met all inclusion criteria, and a third author resolved any discrepancies. The information extracted from each study included sample size, participants' characteristics, experimental design and procedure, neuromuscular performance measurements taken, and main morning vs. afternoon outcomes.

### 2.4 Statistical analysis

The collected data was presented as either the mean ± standard deviation (SD) or mean (SD). Meta-analysis was performed by comparing each dependent variable between morning (7:00–10:00 h) and afternoon/evening conditions (16:00–20:00 h) using Review Manager software (RevMan, version 5.4; The Cochrane Collaboration, Copenhagen, Denmark) to perform statistical analyses. The random effects model was chosen because of the small sample size and the expected heterogeneity among the chosen studies. The study heterogeneity was explored using the I2 static and interpreted as “low” if <50%, “moderate” if I2 is between 50% and 75%, and “high” if I2 is >75%. The effect size of comparison for each dependent variable was determined by Hedgés to reduce the potential bias associated with small sample size. For all analyses, the statistical significance threshold was set at *p* < 0.05.

## 3 Result

### 3.1 Search results

**Figure 1:**
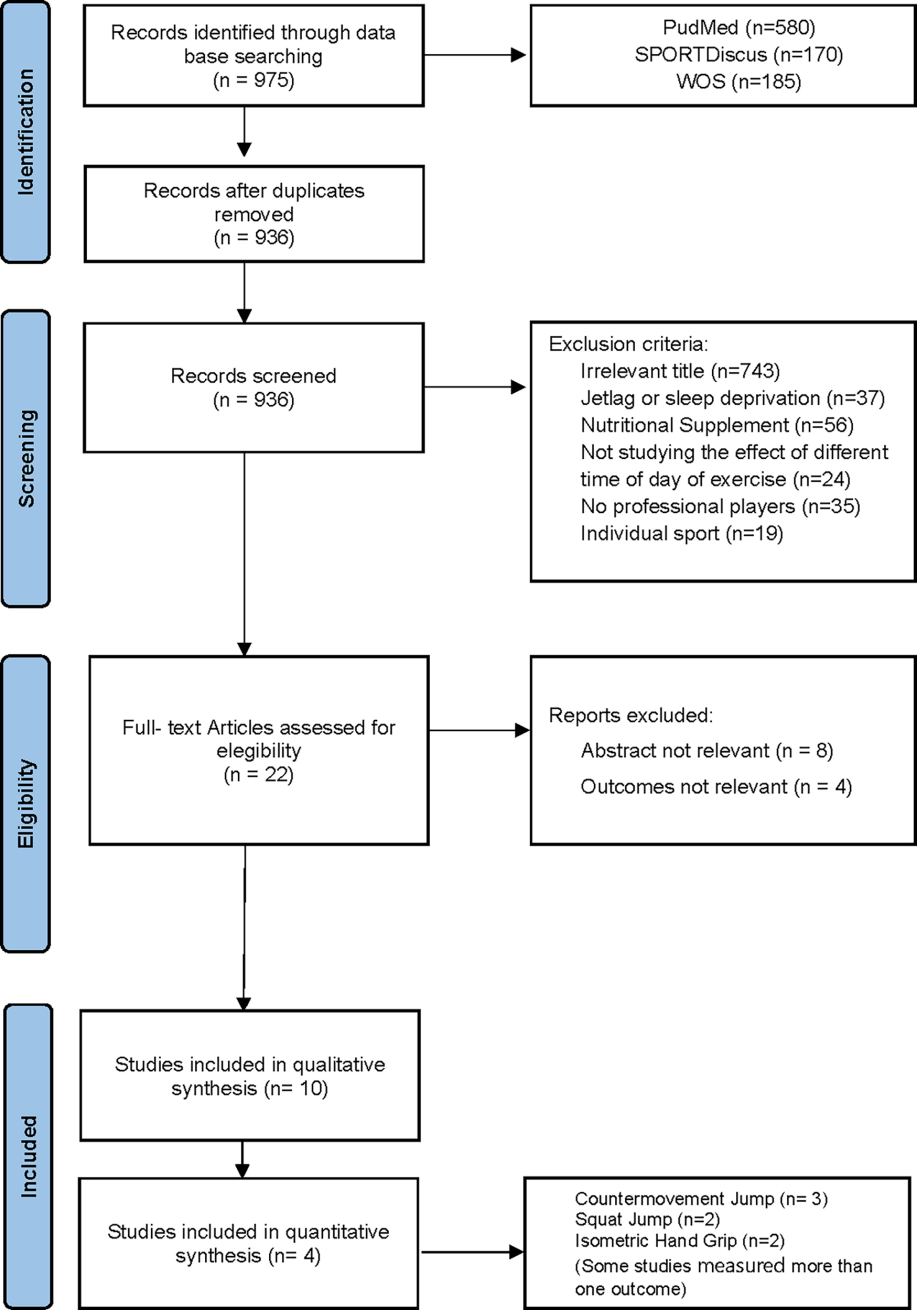
PRISMA flow diagram of the literature screening.

The databases search identified 975 potential full-text research articles. After eliminating duplicate articles and those that did not align with the exclusion and inclusion criteria, 22 articles remained and were selected for full-text screening. Another 12 studies were removed after full-text screening because the (a) abstract were not relevant (b) outcomes were not applicable. Finally, 10 studies were selected for the final review and 5 for the meta-analysis. The flow chart shown in Figure 1 provides the results of the literature search.

### 3.2 Characteristics of included studies and methodological quality

Table 1 outlines the experimental characteristics of 10 selected studies that included 163 healthy professional or semi-professional team sport athletes, aged between 17 and 28 years, being approximately 73% males and 27% females (22, 23, 27–34). All studies employed a similar randomized, crossover experimental design, in which the same participants underwent an experimental trial on at least two occasions, once in the morning (AM) and once in the evening (PM), with the exception of one retrospective study (Heishman et al., 2017) that analyzed previously collected data to evaluate performance. The testing periods for AM and PM trials ranged from 7.00–10.00 and 16.00–22.00 h, respectively. The exercise protocols implemented in these studies were designed to assess the influence of time-of-day on neuromuscular performance (Figure 1).

**Table 1:** Effects of time-of-day on neuromuscular performance in professional elite team players.

Study	Participants	Time of day	Design	Main Outcome	Morning	Evening	Key findings
Heishman et al. (27)	10 Elite male NCAA Division 1 basketball player (20.9 ± 6 1.2 years)	7.00–9.00 h vs. 13.45–16.00 h	Retrospective analysis	Countermovement Jump height (cm)* Countermovement Jump power (W)*	58.3 6 ± 1.4 6,378.0 ± 131.2	61.1 ± 61.9 6,622.1 ± 172.0	CMJ (*p* = 0.008) and Power (*p* = 0.004) were significantly reduced in the morning compared with the afternoon.
Pavlovic et al. (23)	16 elite male handball (25.4 ± 5.8 years)	8.00–9.30 h vs. 18.00–19.30 h	Randomized and cross-over	Zig-zig test (s)* 5 m sprint (s)* 10 m sprint (S)* 20 m sprint (s)* Countermovement Jump height (cm)* Squat Jump height (cm)* Countermovement Jump with arm Swing height (cm)*	4.55 ± 0.16 1.19 ± 0.10 2.03 ± 0.13 3.50 ± 0.25 32.11 ± 4.33 30.54 ± 4.12 38.00 ± 4.52	4.28 ± 0.16 1.06 ± 0.07 1.80 ± 0.09 3.18 ± 0.19 35.75 ± 4.99 33.52 ± 4.09 42.66 ± 4.58	Evening measurements revealed significant increases in jump height for the countermovement jump (CMJ) without (*p* < 0.001) and with arm swing (*p* < 0.001), as well as for the squat jump (*p* < 0.001). Significant improvements were also observed in zig-zag test performance (*p* < 0.001) and sprint times for 5-m, 10-m, and 20-m distances (*p* < 0.001).
Ünver & Atan (28)	20 male soccer players (22.20 ± 3.14 years)	9.00 h vs. 14.00 h vs. 19.00 h	Randomized, counter-balance and cross-over	Wingate Anaerobic Power Test (W/Kg)	10.79 ± 1.06	11.30 ± 1.19	No significant differences were found in peak power at 19.00 compared to 9.00 or 14.00 h (*p* > 0.05).
Martin-López et al. (29)	15 female volleyball players (Superliga 2) (22.3 ± 7.2 years)	9.00 h vs. 19.00 h	Randomized, counter-balance and cross-over	Standing spike test (km h-1)* SEBT posterolateral NO-DOM (cm) Modify Agility *T*-test (s)* Countermovement Jump (cm) Squat Jump (cm) Spike Jump (cm) Isometric handgrip (*N*)	64.2 ± 48 71.8 ± 6.0 5.8 ± 0.3 30.8 ± 4.4 28.1 ± 3.6 40.2 ± 4.0 288.8 ± 56.8	67.1 ± 4.9 75.4 ± 6.5 5.7 ± 0.3 31.0 ± 4.7 28.2 ± 6.6 40.0 ± 4.2 290.9 ± 47.0	Maximal ball velocity in the standing spike test was significantly higher in the evening than in the morning (*p* = 0.002). Additionally, the time to complete the modified *T*-test was lower in the evening compared to the morning (*p* = 0.049).
Pullinger et al. (30)	20 highly motivate male athletes (21.0 ± 2.2 years)	7.30 h vs. 17.30 h	Randomized, counter-balance and cross-over	Repeated sprint ability Distance covered (m)* Average power (W)* Peak velocity (W) Average velocity (Km h-1)*	14.1 ± 1.5 2,353 ± 288 19.8 ± 1.2 16.7 ± 1.8	15.2 ± 1.6 2,537 ± 286 20.4 ± 1.4 18.0 ± 1.9	Distance covered (*p* < 0.001), average power (*p* = 0.001), peak velocity (*p* = 0.001), average velocity (*p* < 0.001) were significantly higher in the evening compared to the morning
Mhenni et al. (31)	15 female Tunisian premier female team handball league (age 20.1 ± 1.5 years)	7.00–8.30 h vs. 17.00–18.30 h	Randomized and cross-over	Isometric handgrip (kg)* Ball-throwing velocity (km.h−1)* Modify Agility *T*-test (s)* Sprint Time (s)* Countermovement Jump (cm)	28.53 ± 6.34 65.7 ± 7.1 7.61 ± 0.64 6.050 ± 0.278 22.56 ± 3.50	33.46 ± 6.74 68.8 ± 4.7 6.68 ± 0.48 5.568 ± 0.071 23.40 ± 3.29	Isometric handgrip (*p* < 0.001), ball-throwing velocity (*p* = 027), and modify Agility *T*-test (*p* < 0.001) performance decreased in the morning compared to the afternoon/early evening. RSSJA, STbest (*p* < 0.001) and STmean (*p* < 0.001), performance were higher in the evening than in the morning.
Moncef Cherif et al. (32)	22 elite male handball players (Tunisian Pro League champion)	8.00 h vs. 18.00 h	Randomized and cross-over	Ball-throwing velocity (m.s-1)* 1RM BH Squat (Kg)* Countermovement Jump (cm)* Repeated sprint ability (s)	22.86 ± 2.18 247.95 ± 33.07 32.97 ± 3.85 6.13	26.21 ± 2.21 272.68 ± 45.05 34.46 ± 4.09 6.05	Significant diurnal variations were observed in the performances of the entire population in BTV (*p* < 0.001), 1-RM BH squat test (*p* < 0.001), and the CMJ test (*p* < 0.01).
Mhenni et al. (22)	15 female team handball players (20.1 ± 1.5 years)	7.00–8.30 h vs. 17.00–18.30 h	Randomized and cross-over	Isometric handgrip (kg)* Ball-throwing velocity (km.h−1)* Modify Agility *T*-test (s)* Countermovement Jump (cm)	28.53 ± 6.34 65.7 ± 7.1 7.61 ± 0.64 22.56 ± 3.50	33.46 ± 6.74 68.8 ± 4.7 6.68 ± 0.48 23.40 ± 3.29	The best performances of HG (*p* = 0.0013), BTV (*p* = 0.027) and MAT (*p* < 0.001) were recorded in the evening compared to the morning.
West et al. (33)	16 Elite male professional rugby union players (21 ± 4 years)	10.00 h vs. 17.00 h	Randomized and cross-over	Countermovement Jump (W)*	5,248 ± 366	5,413 ± 361	Peak power output increased from morning compared to evening (*p* < 0.001)
Hammouda et al. (34)	15 male football players in first division Tunisian league (17.3 ± 0.3)	7.00 h vs. 17.00 h	Randomized and cross-over	Yo-Yo intermittent recovery test (m)*	1,736.64 ± 533.48	2,043.64 ± 533.5	The total distance covered during the Yo-Yo test significantly increased in the evening compared to the morning (*p* < 0.01)

cm, centimeters; *N*, newtons; Kg, kilograms; W, watios; S, seconds; m, meters; CMJ, Countermovement Jump; RSSJA, Repeated shuttle-sprint; ST, Sprint time; BTV, Ball-throwing velocity; 1RM BH squat test, One repetition maximum back half squat at 90; MAT, Modify Agility *T*-test.

* Statistically significant compared to morning session *p* < 0.05.

### 3.3 Qualitative systematic review

Nine used a longitudinal experimental randomized cross-over study (Cherif et al., 2022; Hammouda et al., 2013; Mhenni et al., 2017, 2021; Pavlovic et al., 2018; Pullinger et al., 2014; Martin-López et al., 2022; Ünver & Atan, 2021; West et al., 2014) and one was a retrospective analysis (Heishman et al., 2017). Different neuromuscular tests were performed at various times throughout the day in the 10 studies selected. In five studies the time-of-day effect was assessed in vertical jumping capacity (23, 27, 29, 32, 33). A sample of 79 team-sports athletes, comprising 15 females and 64 males, realized different vertical jumping tests (i.e., squat and countermovement jump) in two different time-of-day periods: morning (7:30–9:00) and evening (17:00–20:00). The results of a pooled analysis of our meta-analysis indicated a diurnal variation, with superior performance in vertical jumping capacity in the afternoon compared to morning values in countermovement jump (5%), but not in the squat jump (5%). Isometric handgrip strength was measured in three of the 10 articles selected (22, 29, 31). Participants (45 females) performed an isometric handgrip strength with the dominant limb test in a neutral position, and the pooled analysis of the studies selected reported no significant differences in the afternoon comparing with morning values (9%) (22, 29, 31). Regarding agility capacity was analyzed in four of the 10 studies (22, 23, 29, 31). Team sports athletes (45 female and 16 male) carried out different validated agility tests (i.e., modified agility *t*-test, zig-zig test). Consistent with earlier findings, the team sports ssqathletes demonstrated enhanced performance in the afternoon tests compared to the morning values (7%). Among the studies reviewed, four specifically investigated the impact of time-of-day on movement speed in short distances (i.e., 0–30 m). Each of these studies examined how neuromuscular metrics varied between morning and afternoon sessions, offering insights into the optimal times for peak athletic performance (23, 30). Both studies were with 36 team sports athletes analyzing velocity in short distances (i.e., 5-m, 10-m, 20-m and all-out 30s sprints) to assess the speed of athlete movement and demonstrated consistent improvements in performance during late afternoon and early evening. Finally, the only study (15 males) that measured muscular endurance found no significant differences between the performance obtained in the morning and the results obtained in the late afternoon and early evening (34).

### 3.4 Assessment of risk of bias

**Figure 2:**
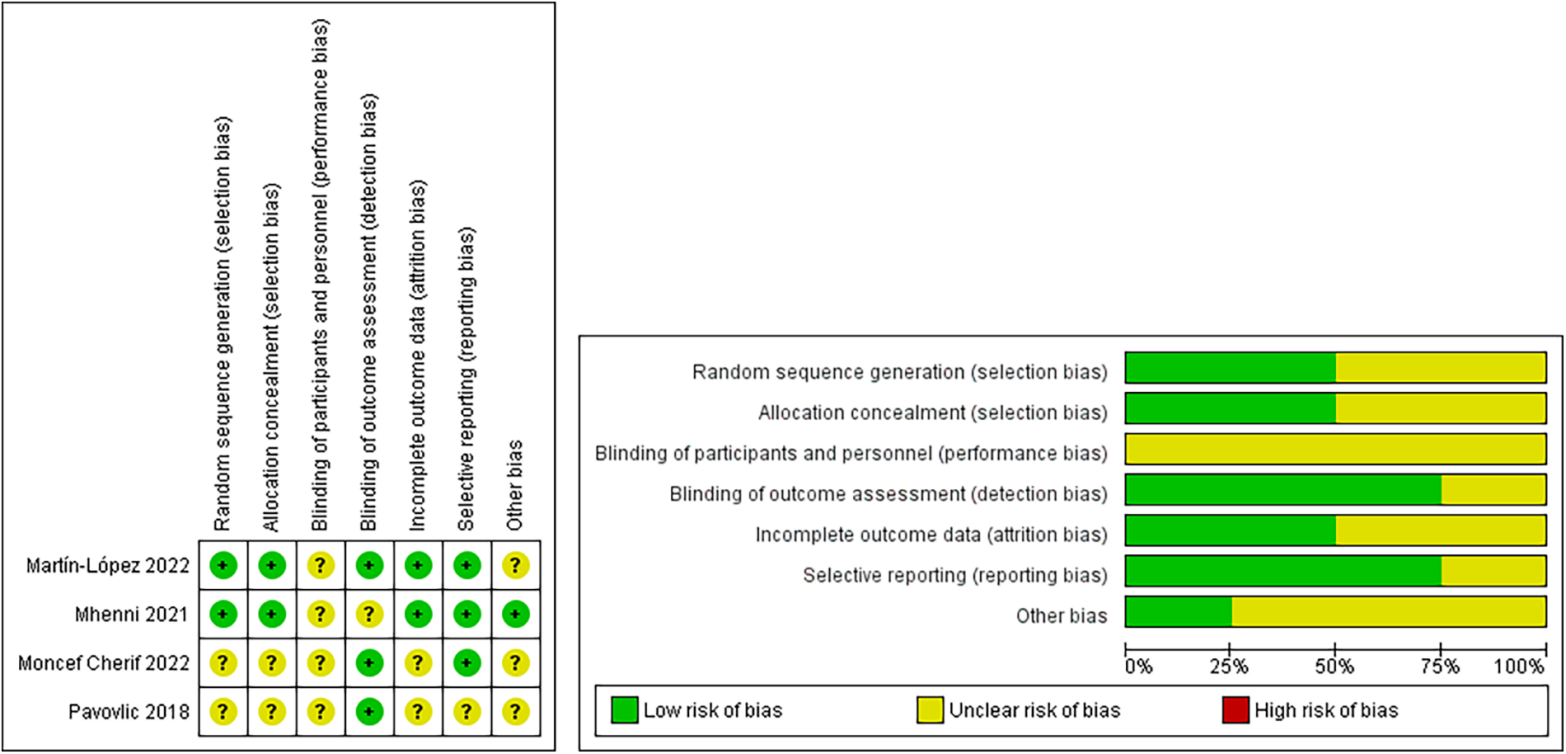
Risk of bias summary.

The Cochrane Collaboration's tool for assessing risk of bias (RoB) was used to evaluate the study risk of bias within the included randomized controlled trials (35). The risk of selection bias, related to a lack of random sequence generation and allocation concealment, was low in two studies using random group allocation (22, 29). Three studies used random allocation, but the method was unclear (23, 32, 33). The methodology for blinding participants, personnel, and outcome assessors was not clearly reported in any of the studies (22, 23, 29, 32, 33), whereas in three studies, only the outcome assessment was blinded (23, 29, 32). The risk of attrition bias was deemed unclear in only 2 studies because they lost participants in the follow-up that were needed for analysis (23, 32). The risk of reporting bias was unclear in only 1 study of the meta-analysis (23) (Figure 2).

### 3.5 Heterogeneity

Heterogeneity was assessed using the I^2^ statistic, interpreted according to standard thresholds. For metrics with moderate to high heterogeneity (I^2^ > 50%), such as agility (I^2^ = 85%), a random-effects model was applied to mitigate the influence of differences between studies. This approach allowed us to estimate a conservative and generalizable average effect while acknowledging the inherent variability in study designs and populations. The high heterogeneity observed in agility metrics, for instance, can be attributed to differences in test protocols (e.g., modified agility *T*-test vs. zig-zag test) and sample characteristics, including variations in age, sex, and competition level (professional vs. semi-professional athletes). By employing a random-effects model, we accounted for these discrepancies, ensuring that the results reflect the variability across studies while maintaining robustness in the meta-analysis conclusions.

We evaluated the heterogeneity of the four studies included in the meta-analysis. Statistical heterogeneity was calculated for continuous variables (squat and countermovement jump, isometric handgrip strength and agility values). In the three studies that analyzed the agility capacity, heterogeneity was high (I2 = 85%; *p* = 0.001) (22, 23, 29). Heterogeneity was also high for the two studies including isometric handgrip strength (I2 = 57%; *p* = 0.13) (22, 29). Heterogeneity was low for three studies that measured the countermovement jump (I2 = 10%; *p* = 0.33) (23, 29, 32) and for the two studies that included squat jump (I2 = 29%; *p* = 0.23) (23, 29). No studies were excluded due to a high risk of bias that could influence the presented heterogeneity.

### 3.6 Quantitative meta-analysis

**Figure 3:**
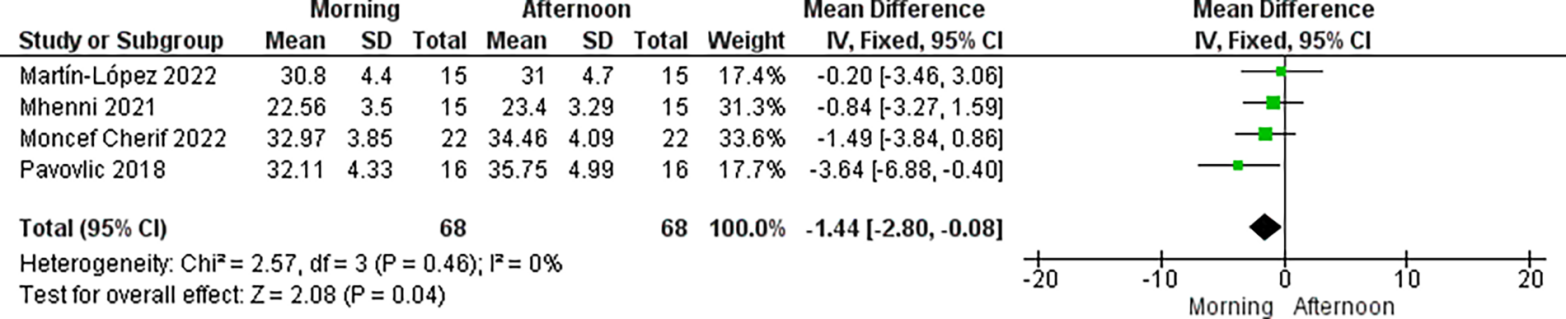
Forest plot of changes in countermovement jump performance in team sports athletes comparing morning vs. afternoon.

**Figure 4:**
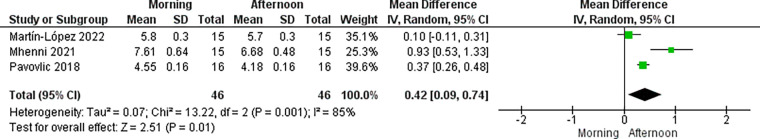
Forest plot of changes in agility performance in team sports athletes comparing morning vs. afternoon.

A total of four studies involving 68 athletes (38 males and 30 females) were included in the quantitative meta-analyses. For statistical analysis, data was categorized according to two time-of-days: morning (AM, 7:00–10:00 h) and late afternoon or early evening (PM, 16:00–20:00 h). These categories were applied to each of the following continuous outcomes: countermovement jump (4 studies, *n* = 68 subjects) (22, 23, 29, 32), squat jump (2 studies, *n* = 31 subjects) (23, 29), isometric handgrip strength (2 studies, *n* = 30 subjects) (22, 29) and agility test (3 studies, *n* = 46 subjects) (22, 23, 29). Countermovement jump presented lower values in the AM than in the PM (mean difference, −1.44; 95% CI – 2.80 to−0.08; *p* = 0.04) (Figure 3) and MAT also reported higher values in the PM comparing to AM difference (0.42; 95% CI 0.09 to 0.74; *p* = 0.01) (Figure 4).

**Figure 5:**
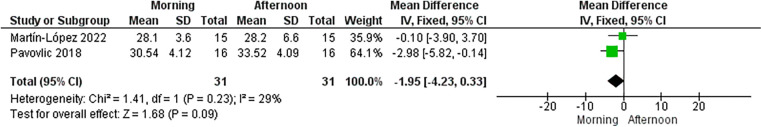
Forest plot of changes in squat jump performance in team sports athletes comparing morning vs. afternoon.

**Figure 6:**
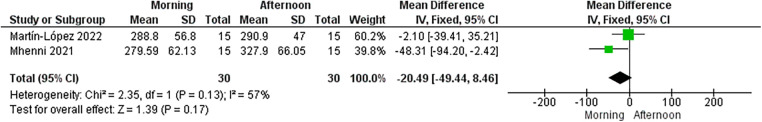
Forest plot of changes in isometric strength performance in team sports athletes comparing morning vs. afternoon.

No differences were reported between the time-of-day conditions in squat jump (mean difference,−1.95; 95% CI −4.23 to 0.33; *p* = 0.09) (Figure 5) as same as in isometric handgrip strength (mean difference, −20.49; 95% CI – −49.44 to 8.46; *p* = 0.17) (Figure 6).

## 4 Discussion

The aim of this systematic review and meta-analysis was to investigate the diurnal variation in neuromuscular performance of professional or semi-professional team-sports athletes. Our findings indicate a significantly higher performance in the late afternoon and early evening in vertical jumping capacity (i.e., countermovement jump) and agility values compared to morning while no statistically significant differences were obtained in isometric handgrip strength and squat jump, in the meta-analysis that included a population of 68 athletes (38 males and 30 females).

Vertical jump performance is a critical factor in the success of athletes in team sports, as it plays a significant role in various athletic disciplines (Suchomel et al., 2016). This performance is typically evaluated using tests such as the countermovement jump and squat jump, which allow for the measurement of lower limb strength and power output. In our study, three of the four studies included in the meta-analysis showed higher mean vertical jump capacity in the countermovement jump during the late afternoon and early evening compared to the morning (22, 23, 32) while one did not report diurnal differences between protocols (29). Thus, according to our data, there was a consistent pattern despite the limited number of studies that support the fact that countermovement jumps capacity test peaks in the late afternoon and early evening compared to the morning values. Referring to the squat jump, two of the three studied included in this meta-analysis consistently showed higher mean vertical jump capacity not reaching statistical significance. These differences between morning to afternoon and late evening values in vertical jump capacity could be attributed to the typical pattern of increased core temperature that occurs late in the afternoon or early evening, a result of transient changes in heat generation and loss throughout the day (36). This increase in body temperature may induce a passive warm-up effect (37), enhancing metabolic nerve conduction velocity (38) and affecting muscle contractile function (39).

Agility is one of the determining factors in neuromuscular performance in team sports athletes, as comprehend the ability to quickly accelerate, decelerate, change direction and maintain a proper balance (40). According to several studies, agility performance tends to show a peak during the late afternoon and early evening hours. In our study, two of three studies included in this meta-analysis showed an enhancement in agility performance in afternoon-evening compared to morning protocols (23, 31), while the remaining study did not observe this difference (29). Recent findings suggest that elite or professional athletes may be more influenced by time-of-day compared to athletes at other level (e.g., semi-professional athletes) due to elite levels presented higher neural activation and muscular strength/power values that could be more affected by temperature changes and neural recruitment patterns along the day affecting specially in elite athletes comparing to amateur or recreational athletes (41).

Isometric handgrip strength is frequently utilized as an indicator of enhanced force production capacity across both upper and lower body musculature (i.e., weightlifting, paddling), as well as proficiency in executing intricate movements (i.e., rackets or stick skills) (42). Therefore, isometric handgrip is a cheap and easy test to implement among team sports athletes and can be related to various specific actions involving the upper body actions (e.g., blocking, hitting) that occurs in different teams' sports disciplines (Cronin et al., 2017). In our study, one of the two studies included in this meta-analysis showed statistical diurnal differences (31), while another study did not report differences (29). The differences reported between these studies could be attributed to the specific demands of the team sports disciplines measured (i.e., handball vs. volleyball). Isometric strength demands are notably elevated in handball due to the continuous need for catching, passing, and controlling the ball, which occurs frequently during training and competitions, compared to volleyball where the contact with the ball in hitting, passing, digging or blocking actions occurs in thousandths of a second. Additionally, as previously mentioned, the different levels of competition (i.e., professional vs. semi-professional) may also impact the outcomes presented. The findings of this study reinforce the importance of considering time-of-day variations in neuromuscular performance when designing training and competition schedules for team sport athletes. Beyond the physical aspects, the psychological readiness of athletes also plays a critical role in optimizing performance (12, 13). These findings suggest that coaches should adopt a dual approach, considering both physical and psychological factors when scheduling training sessions. By aligning training schedules with both the physiological and psychological needs of athletes, practitioners can ensure a more comprehensive optimization of performance.

Our systematic review and meta-analysis have several limitations that should be mentioned to enhance its applicability to real sports context scenarios. First limitation of this systematic review and meta-analysis is the scarcity of experimental studies examining the relationship between diurnal variations and neuromuscular performance in professional or semi-professional team sport athletes and that the small sample sizes increase the risk of statistical error and may not capture the full variability in neuromuscular performance across athletes, especially when considering individual differences such as chronotype or sex. Second, is the heterogeneity in study designs, methodologies, and outcome measures across the included studies. Third, the meta-analysis included both male and female participants, which, while necessary due to limited evidence, may have obscured potential gender-specific differences in time-of-day effects on performance. Future research should investigate gender-specific physiological and neuromuscular responses to time-of-day variations to develop tailored performance and training strategies. Fourth, individual differences in chronotype, such as morningness or eveningness preferences, were not fully accounted for in this study. Chronotypes influence performance, with morning-oriented athletes excelling earlier in the day and evening-oriented athletes performing better later. Future research should explore these variations to develop personalized training and competition schedules that align with athletes' natural rhythms. This variability makes it challenging to directly compare and combine the results, leading to potential inconsistencies and reduced generalizability.

## 5 Conclusion

Neuromuscular performance in professional and semi-professional team-sport athletes is influenced by the time of day, with higher performance levels observed in vertical jump capacity and agility performance during the afternoon and evening (4–8 PM) compared to the morning time (7–10 AM).

## Data Availability

The raw data supporting the conclusions of this article will be made available by the authors, without undue reservation.
